# Detection of *Neisseria meningitidis* in saliva and oropharyngeal samples from college students

**DOI:** 10.1038/s41598-021-02555-x

**Published:** 2021-11-30

**Authors:** Willem R. Miellet, Rob Mariman, Gerlinde Pluister, Lieke J. de Jong, Ivo Grift, Stijn Wijkstra, Elske M. van Logchem, Janieke van Veldhuizen, Marie-Monique Immink, Alienke J. Wijmenga-Monsuur, Nynke Y. Rots, Elisabeth A. M. Sanders, Thijs Bosch, Krzysztof Trzciński

**Affiliations:** 1 Centre for Infectious Disease Control Netherlands, National Institute for Public Health and the Environment (RIVM), Bilthoven, The Netherlands; 2 University of Applied Sciences Utrecht, Utrecht, The Netherlands; 3 Department of Pediatric Immunology and Infectious Diseases, Wilhelmina Children’s Hospital, University Medical Center Utrecht, Utrecht, The Netherlands

**Keywords:** Respiratory tract diseases, Bacteriology

## Abstract

Carriage of *Neisseria meningitidis* is an accepted endpoint in monitoring meningococcal vaccines effects. We have assessed *N. meningitidis* and vaccine-type genogroup carriage prevalence in college students at the time of MenACWY vaccine introduction in the Netherlands, and evaluated the feasibility of saliva sampling for the surveillance of carriage. For this, paired saliva and oropharyngeal samples collected from 299 students were cultured for meningococcus. The DNA extracted from all bacterial growth was subjected to qPCRs quantifying meningococcal and genogroup-specific genes presence. Samples negative by culture yet positive for qPCR were cultured again for meningococcus. Altogether 74 (25%) of students were identified as meningococcal carrier by any method. Sixty-one students (20%) were identified as carriers with qPCR. The difference between number of qPCR-positive oropharyngeal (n = 59) and saliva (n = 52) samples was not significant (McNemar’s test, *p* = 0.07). Meningococci were cultured from 72 students (24%), with a significantly higher (*p* < 0.001) number of oropharyngeal (n = 70) compared with saliva (n = 54) samples. The prevalence of genogroups A, B, C, W, and Y was none, 9%, 1%, 1% and 6%, respectively, and 8% of students carried MenACWY vaccine-type genogroup meningococci. Saliva is easy to collect and when combined with qPCR detection can be considered for meningococcal carriage studies.

## Introduction

*Neisseria meningitidis* (meningococcus) is a commensal of the human upper respiratory tract (URT) and a major cause of invasive bacterial disease^1^. Adolescents are at increased risk of invasive meningococcal disease (IMD)^2^. Following an outbreak of serogroup W IMD in the Netherlands in the fall of 2018, a monovalent conjugate polysaccharide vaccine targeting serogroup C (NeisVac-C, Pfizer) was replaced in the National Immunization Program with a tetravalent conjugated polysaccharide vaccine (Nimenrix, GlaxoSmithKline) targeting serogroups C, A, W, and Y^3^. Initially, the MenACWY vaccine was given only to 14-months-old children, but since 2019 it is also offered to 14 year olds^4^. Conjugated vaccines not only protect against disease but also reduce carriage of vaccine-type (VT) strains^5^. Since the prevalence of meningococcal carriage is reported to peak in adolescents and young adults, vaccination in teenagehood is expected to induce herd protection across the population^2^. Effects of conjugated polysaccharide vaccines can be monitored via surveillance of carriage^6^. For this, reliable and efficient detection methods for meningococcus are required.

Oropharyngeal samples have been widely used to detect meningococcal carriage as it has been reported that oropharyngeal samples are more sensitive than nasal or nasopharyngeal samples^7^. While a role for saliva in meningococcal transmission has been implicated in multiple studies^8–16^, and closely-related *Neisseria* species are often cultured from saliva^17^, few studies have tested saliva for meningococci^18–21^. In general, saliva is described to be poorly suited for meningococcal detection^19^. Unlike oropharyngeal and nasopharyngeal swabs, saliva sampling is noninvasive, and oral fluids can be easily self-collected.

Our first objective was to establish a pre-vaccination baseline for VT carriage prevalence among college students as it will allow us to assess the impact of MenACWY vaccine in the Netherlands in the future. The second objective was to investigate the use of saliva samples to monitor meningococcal carriage.

## Materials and methods

### Ethics statement

The study protocol was reviewed by the Centre for Clinical Expertise at the RIVM. Since procedures were considered non-invasive, and participants were anonymized, the study was considered outside the ambit of the WMO (Medical Research Human Subjects Act, http://www.ccmo.nl). Consequently, the committee approved the consent procedure and granted a waiver for further ethical review. The study was conducted in accordance with the World Health Medical Association 1966 Declaration of Helsinki and the EU rules of Good Clinical Practice.

### Study design and sample collection

In the fall of 2018, saliva and oropharyngeal swabs were collected from college students of Hogeschool Utrecht (n = 300). After signing informed consent, students self-collected saliva by spitting 1 ml into a 15 ml tube (Greiner, Kremsmünster, Austria). Next, a study nurse swabbed student’s posterior pharyngeal wall with a nylon swab (FLOQSwabs, COPAN, Brescia, Italy) to collect an oropharyngeal sample. Immediately after collection, saliva (approximately 50 µl) and oropharyngeal swab were used to inoculate Neisseria Selective Medium PLUS agar plates (NS-agar, Oxoid, Badhoevedorp, the Netherlands) and within 20 min plates were placed in a 37 °C, 5% CO_2_ incubator. Once all samples have been collected, cultured plates were transported at room temperature to the laboratory.

### Meningococcal carriage detection using culture

Upon arrival, NS-agar cultures were incubated for up to two days at 37 °C and 5% CO_2_. On both days cultures were screened for presence of meningococcus-like colonies (grey, round and smooth colonies with convex shape). When found, 1–3 colonies were re-plated on Columbia Blood agar (CBA, bioTRADING Benelux B.V., Mijdrecht, the Netherlands) and tested for species identification using Matrix-assisted Laser Desorption/Ionization Time-of-Flight mass spectronomy (MALDI-ToF, Bruker Daltonik GmbH, Bremen, Germany). Separately for oropharyngeal and saliva samples, a single isolate with a score ≥ 2.0 for *Neisseria meningitidis* (database BDAL V8.0.0.0 + SR1.0.0.0, Bruker Daltonik) was stored at − 70 °C in Brain Heart Infusion (BHI, Oxoid) supplemented with 0.5% Yeast Extract (YE, Oxoid) and 10% glycerol. NS-agar cultures displaying any microbial growth were harvested into 2 ml of Todd–Hewitt Broth (Oxoid) supplemented with 0.5% YE and 10% glycerol. These harvests were considered to be culture-enriched for meningococci, and 0.7 ml of it stored at − 70 °C.

### Detection of meningococcal DNA with qPCR

DNA was extracted from 100 µl of harvest of culture-enriched samples using DNeasy Blood and Tissue kit (Qiagen, Hilden, Germany) as previously described^22^. DNA eluted into 100 µl sample volume was tested in quantitative-PCRs (qPCRs) using primers and probes (Eurogentec, Seraing, Belgium) targeting sequences within *metA,* a gene encoding for a periplasmic protein, and a capsule transporter gene *ctrA*^23,24^. The qPCRs were conducted using Probes Master 480 (Roche) mastermix, primers and probes concentrations are listed in Table S1, with 5.5 µl of DNA sample used in 12.5 µl reaction volumes. The qPCR assays were conducted on LightCycler480 (Roche) with programme as described in Table S2. A tenfold serial dilution of DNA from a meningococcal strain (Table S3) was used as standard curve. C_T_ thresholds for positivity were determined with Youden index calculated using Receiving operating characteristic (ROC) curve analysis^25^.

### Recovery of live meningococcu*s* from culture-enriched samples

To test whether lower sensitivity of conventional diagnostic culture could account for differences between qPCR and culture results, culture-enriched samples first classified as negative by culture yet positive by qPCR were revisited to recover viable meningococci. For this second culture guided by qPCR results, CBA plates were inoculated with 100 µl culture-enriched sample in 10^–2^–10^–4^ dilutions, incubated at 37 °C and 5% CO_2_, and screened for meningococcus as described above.

### Genogroup-specific qPCRs

Two microliters of DNA extracted from culture-enriched samples were tested in 12.5 µl of reaction volume in qPCRs targeting genogroups A, B, C, W or Y^24^. Primer and probe concentrations are listed in Table S1. These qPCRs were conducted on a LightCycler480, using SensiFast probe No-ROX mastermix (Bioline, London, United Kingdom) and with programme described in Table S2. Culture-enriched samples were regarded as positive for a genogroup when the C_T_ was lower than the cut-off value set for *metA* and *ctrA*. Control strains are listed in Table S3.

### Genotyping of meningococcal strains

DNA extracted from cultured strains was tested in *metA*, *ctrA* and genogroup-specific qPCRs. Since not all genogroups were covered by qPCRs, a simplified criterium of positivity for *ctrA* was also applied to classify strain as genogroupable.

### Statistical analysis

Data was analyzed using Prism (GraphPad Software, v8.4.1) and R (version 4.0.0). A *p* value of < 0.05 was considered significant. ROC curve analysis was performed using “cutpointr” package^25^, and Cohen’s Kappa (*κ*) was determined in analysis of methods agreement. Youden index values were determined via bootstrapping (n = 1000) on *metA* qPCR data from saliva and oropharyngeal samples to determine the optimal cut-off value for qPCR detection^25^.

## Results

**Figure 1:**
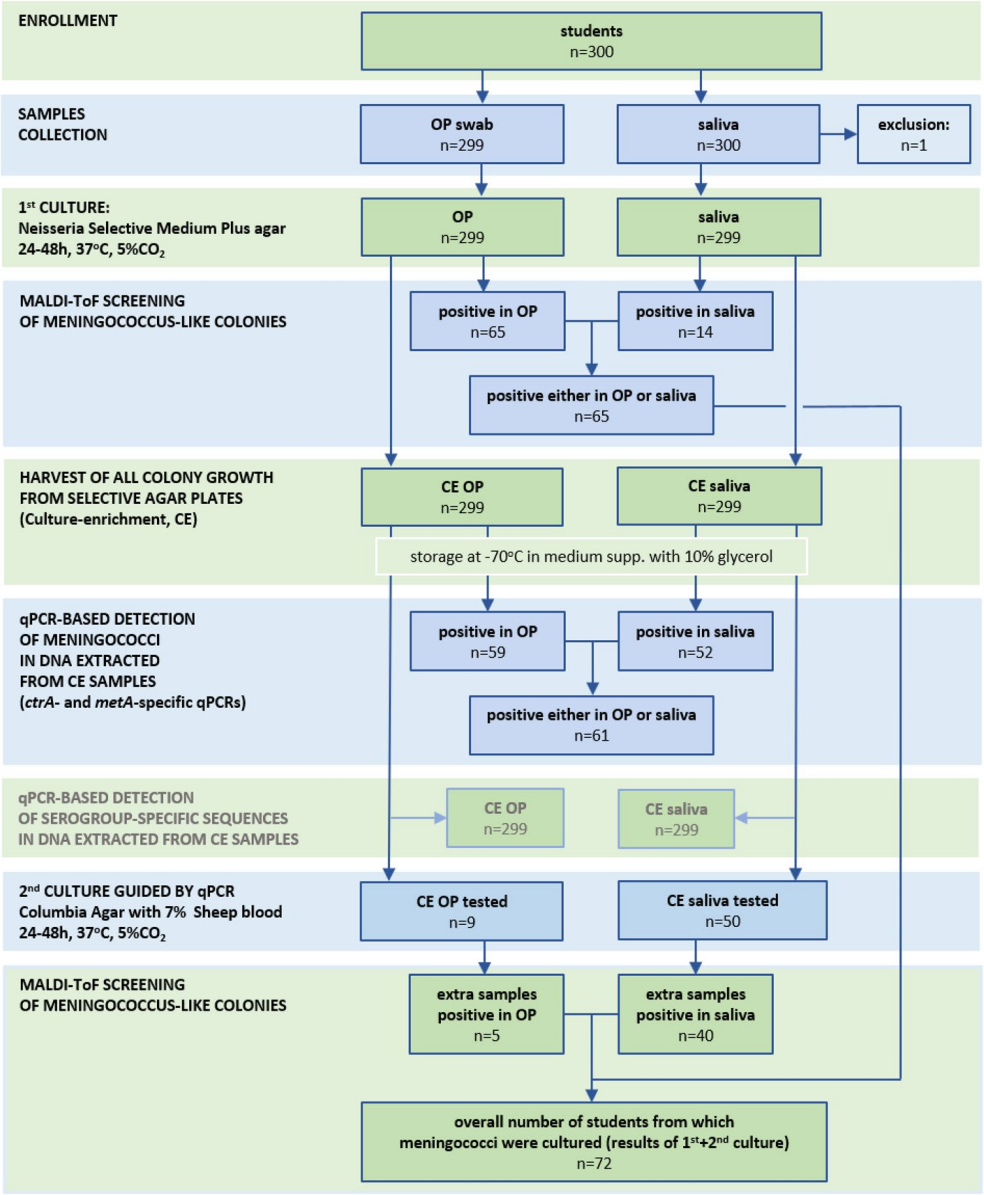
Flowchart depicting the study workflow and results of meningococcal detection using either culture-based or qPCR-based diagnostic methods. *OP* oropharyngeal sample, *CE* culture-enriched sample.

All samples were collected in October and November 2018. Of 300 students that consented to participate, one person refused to have the oropharynx swabbed and was excluded from the study. Paired saliva and oropharyngeal samples from the remaining 299 (61% female; median age 20 years, range 16–28 years) students were analyzed (Fig. 1).

Bacterial strains classified with MALDI-ToF as meningococcus were cultured from 72 students (24% of 299) of which 70 had strains isolated from the oropharynx and 54 from saliva (Table 1). Sixty-five (93%) of 70 oropharyngeal samples positive by culture had meningococcus isolated from the first culture and the remaining five strains were recovered when samples positive by qPCR yet initially culture-negative for meningococcus were revisited. For saliva, the same procedure resulted in fourteen samples positive for meningococcal strains in the first culture (26% of 54) and the remaining 40 in cultures guided by qPCR showing that initial diagnostic cultures displayed vastly reduced sensitivity for saliva when compared with oropharyngeal samples (14 vs. 65 strains cultured from 299 students, McNemar’s test, *p* < 0.0001). The differences also remained significant after qPCR-guided culture (54 vs. 70, *p* < 0.001). Genogroupable meningococci were cultured from 62 students (21% of 299). Here too, the number of culture-positive samples was significantly higher for oropharyngeal swabs compared with saliva (58 vs. 45, *p* < 0.01) (Table 2).

**Table 1:** The accuracy of *Neisseria meningitidis* detection in oropharyngeal and saliva samples collected from 299 students and tested with culture and using molecular methods applied to DNA extracted from culture-enriched samples. Measures of diagnostic accuracy were calculated by comparing the number of individuals positive per method with the overall number of individuals positive for *N. meningitidis* by any method.

Method	Oropharyngeal swab							Saliva						
	Prevalence % (*95% CI*)	PPV %	NPV %	Sensitivity % (*95% CI*)	Specificity % (*95% CI*)	Concordance % (*95% CI*)	*κ*	Prevalence % (*95% CI*)	PPV %	NPV %	Sensitivity % (*95% CI*)	Specificity % (*95% CI*)	Concordance % (*95% CI*)	*κ*
Initial culture	21.7 (*17.4–26.8*)	100	97.0	90.3 (*81.3–95.2*)	100 (*98.3–100*)	97.7 (*95.2–98.9*)	0.93	4.7 (*2.8–7.7*)	100	79.6	19.4 (*12.0–30.0*)	100 (*98.3–100*)	80.6 (*75.7–84.7*)	0.27
qPCR	19.7 (*15.6–24.6)*	98.3	94.2	80.6 (*70.0–88.0*)	99.6 (*97.5–99.9*)	95.0 (*91.9–96.9*)	0.85	17.4 (*13.5–22.*1)	98.1	91.5	70.8 (59*.5–80.1*)	99.6 (*97.5–99.9*)	92.6 (*89.1–95.1*)	0.78
Initial plus qPCR-guided cultures	23.4 (*19.0–28.5*)	100	99.1	97.2 (*90.4–99.2*)	100 (*98.3–100*)	99.3 (*97.6–99.8*)	0.98	17.7 (*13.8–22.5*)	100	92.3	73.6 (*62.4–82.4*)	100 (*98.3–100*)	93.6 (*90.3–95.9*)	0.81

*PPV* positive predictive value, *NPV* negative predictive value, *95% CI* 95% confidence interval, *κ* Cohen’s Kappa where ≤ 0, 0.01–0.20, 0.21–0.40, 0.41–0.60, 0.61–0.80, > 0.81 are interpreted as no agreement, none to slight, fair, moderate, strong, and almost perfect agreement, respectively.

**Table 2:** The accuracy of genogroupable *Neisseria meningitidis* detection in oropharyngeal and saliva samples collected from 299 students and tested with culture and using molecular methods applied to DNA extracted from culture-enriched samples. Measures of diagnostic accuracy were calculated by comparing the number of detected individuals positive per method with the overall number of individuals positive for genogroupable *N. meningitidis*.

Method	Oropharyngeal swab							Saliva						
	Prevalence % (*95% CI*)	PPV %	NPV %	Sensitivity % (*95% CI*)	Specificity % (*95% CI*)	Concordance % (*95% CI*)	*κ*	Prevalence % (*95% CI*)	PPV %	NPV %	Sensitivity % (*95% CI*)	Specificity % (*95% CI*)	Concordance % (*95% CI*)	*κ*
Initial culture	17.7 (*13.8–22.5*)	81.5	97.0	88.3 (*77.8–94.2*)	95.0 (*91.4–97.1*)	93.6 (*90.3–95.9*)	0.81	4.3 (*2.6–7.3*)	92.9	83.5	21.7 (*13.1–33.6*)	99.6 (*97.7–99.9*)	83.9 (*79.4–87.7*)	0.30
qPCR	19.4 (*15.3–24.3*)	98.3	99.2	96.7 (*88.6–99.1*)	99.6 (*97.7–99.9*)	99.0 (*97.1–99.7*)	0.97	17.1 (*13.2–21.7*)	98.1	96.4	85.0 (*73.9–91.9*)	99.6 (*97.7–99.9*)	96.7 (*94.0–98.2*)	0.89
Initial plus qPCR-guided cultures	19.4 (*15.3–24.3*)	82.9	99.1	96.7 (*88.6–99.1*)	95.0 (*91.4–97.1*)	95.3 (*92.3–97.2*)	0.86	14.7 (*11.1–19.2*)	83.0	93.5	73.3 (*61.0–82.9*)	96.2 (*93.0–98.0*)	91.6 (*87.9–94.3*)	0.73

*PPV* positive predictive value, *NPV* negative predictive value, *95% CI* 95% confidence interval, *κ* Cohen’s Kappa where ≤ 0, 0.01–0.20, 0.2–0.40, 0.41–0.60, 0.61–0.80, > 0.81 are interpreted as no agreement, none to slight, fair, moderate, strong, and almost perfect agreement, respectively.

**Figure 2:**
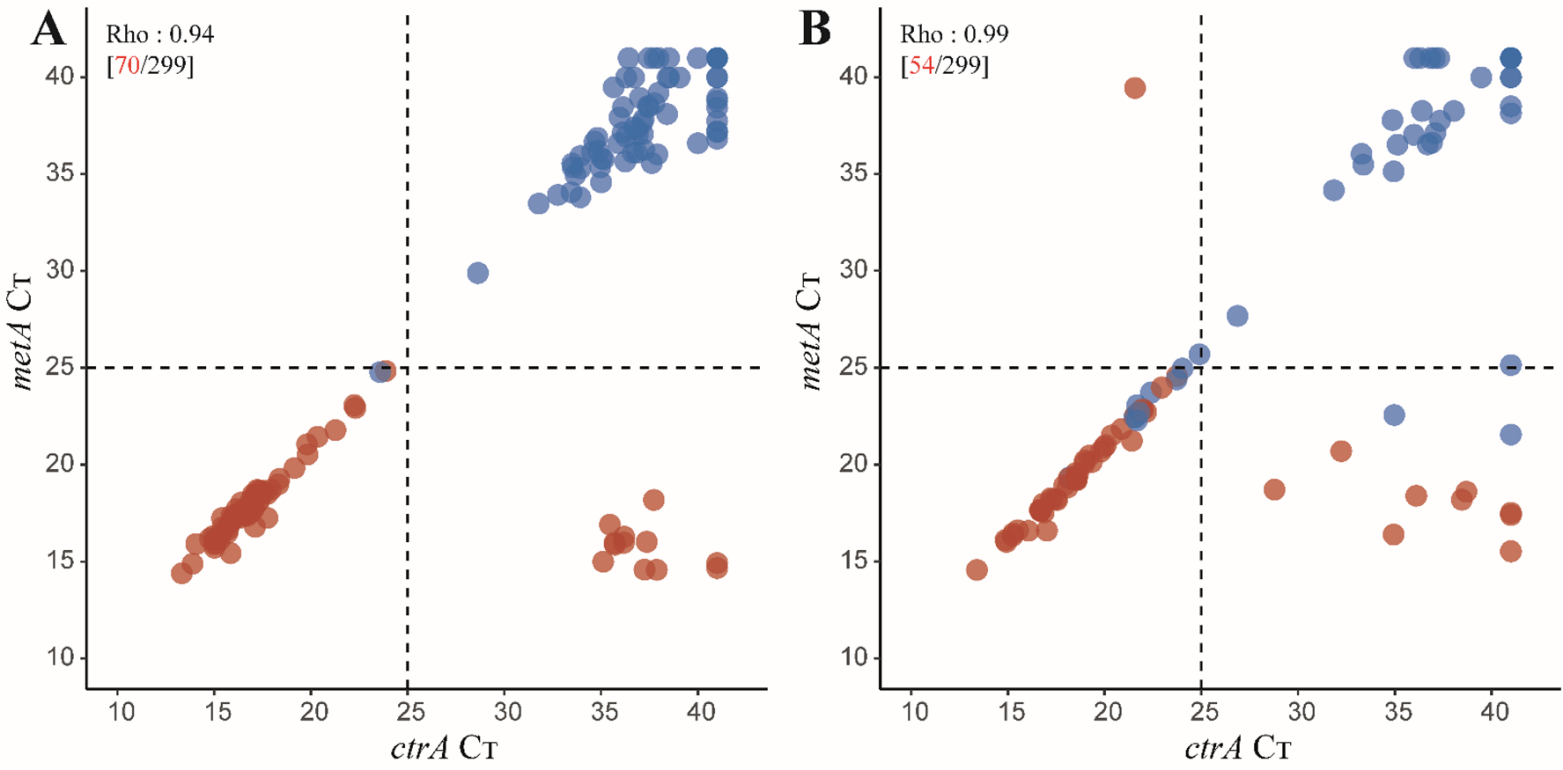
qPCR based detection of *Neisseria meningitidis* versus isolation of live meningococci from oropharyngeal and saliva samples. A scatter plot of the *metA* and *ctrA* qPCR cycle threshold (C_*T*_) values from (**A**) oropharyngeal and (**B**) saliva samples. Each symbol represents an individual sample. Samples with a C_*T*_ > 25 for both *metA* and *ctrA* are considered as positive for meningococcal carriage when tested with molecular methods. In both oropharyngeal and saliva samples, we noted a significant correlation between *metA* and *ctrA* for meningococcus positive samples (Spearman’s test p < 0.0001). Red dots represent samples from which meningococcal strain has been cultured. Blue dots represent samples classified as positive for meningococcus when tested with molecular method but negative by culture. Numbers in brackets depict the number (in black) of all samples and (in red) number of samples from which *N. menigitidis* has been cultured.

**Figure 3:**
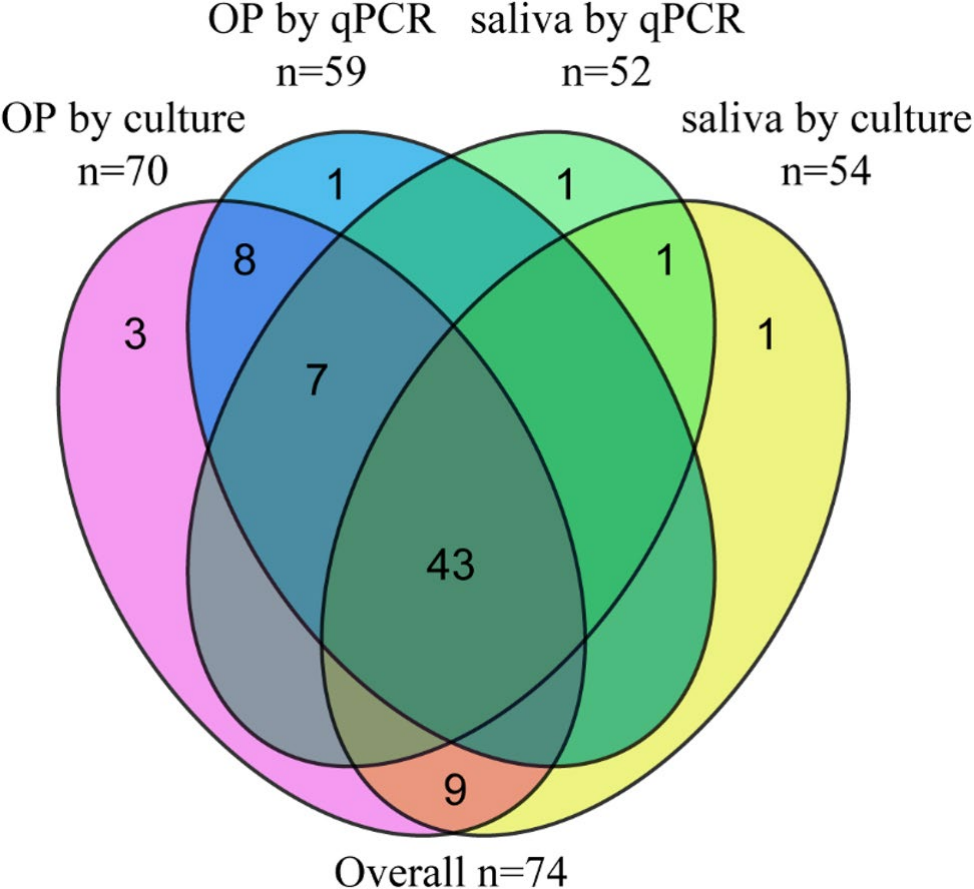
Venn diagram displaying the number of oropharyngeal and saliva samples positive for meningococci based on recovery of live *N. meningitidis* strain from a culture (samples positive by culture, includes qPCR-guided culturing) or when tested with qPCR.

The study criterium for classification of a sample as positive for *N. meningitidis* by qPCR was detection of both *metA* and *ctrA* in DNA extracted from a culture-enriched sample, and was derived by calculating the optimal C_T_ cut-off values by using the Youden index (Table S4). Using qPCR detection we identified 61 students (20% of 299) as a meningococcal carrier. The difference in proportion of carriers detected by qPCR between oropharyngeal samples and saliva samples was not significant (59 or 20% vs. 52 or 17%, McNemar’s test; *p* = 0.0704), both methods showed high agreement (96%; *κ* 0.88). Samples classified as positive for meningococcus by qPCR displayed significant correlation between *metA* and *ctrA*, supporting high specificity of molecular detection (Fig. 2). Detection by culture and by qPCR resulted in 71 (24% of 299) and 62 (21%) students identified as a meningococcal carrier in oropharyngeal and saliva samples, respectively. Altogether 74 (25%) students were identified as a meningococcal carrier and 62 (21%) as carrier of genogroupable meningococci by any method used (Fig. 3).

When comparing methods and specimen types used for detection of meningococcal carriage overall, all evaluated procedures displayed comparable specificity of detection (Table 1) and primarily varied in performance for sensitivity and for positive predictive value (PPV).

The criterium based on both *ctrA* and *metA* was expected to impact negatively the sensitivity of meningococcal carriage detection by qPCR when compared with culture due to the presence of non-genogroupable meningococci that were likely to be *ctrA*-negative. Therefore, we compared methods and specimen types on samples containing genogroupable meningococci (Table 2), which were supposed to be positive for both *ctrA* and *metA*. The PPV and sensitivity of the evaluated methods were highest for detection by qPCR, whereas using saliva samples resulted in decreased negative predictive values (NPV) when compared with oropharyngeal samples. Detection of genogroupable meningococci using qPCR and saliva displayed increased PPV and comparable sensitivity when compared with detection of meningococcus in initial oropharyngeal cultures.

**Figure 4:**
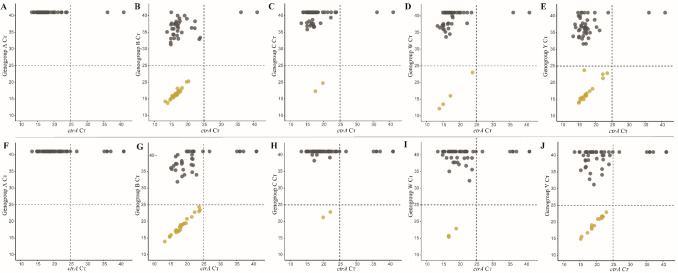
A scatter plot of the *ctrA* and genogroup-specific qPCR cycle threshold (C_*T*_) values. Results are displayed for oropharyngeal (**A**–**E**) and saliva (**F**–**J**) samples. Each dot represent an individual sample. Samples with a C_*T*_ for both ctrA and a particular genogroup below 25 C_*T*_ are considered as positive for that particular genogroup. Yellow dots represent samples classified as positive for a genogroup by qPCR and grey dots as negative for the depicted genogroup. Dashed lines depict the C_*T*_ criterium for meningococcal carriage.

Next, we determined with genogroup-specific qPCRs the prevalence of genogroup A, B, C, W and Y carriage. The specificity of these qPCR assays was tested using culture-enriched samples negative for meningococcal carriage by culture and qPCR and none of the samples negative for *ctrA* generated a signal below 25 C_T_ for a genogroup-specific gene (Fig. S1). Altogether, 51 (83.6%) of 61 students identified as carriers of meningococci with qPCR were positive for any of the genogroups targeted in group-specific qPCRs (Fig. 4). Genogroup-specific C_T_s of almost all samples positive for any of the tested genogroups corresponded strongly to the C_T_ for *ctrA*. The exception was a single oropharyngeal sample for which results were indicative of potential co-carriage of a genogroup Y strain with another *ctrA*-positive meningococcal strain of unidentified group (Fig. 4E). The prevalence of genogroup B (8.7% of 299) and Y (6.4% of 299) was highest while genogroups C (0.7% of 299) and W (1.3% of 299) were less prevalent. None of the samples were positive for genogroup A. MenACWY VT genogroups accounted for 8.4% (95% CI 5.7–12.1) carriage prevalence or 41.0% of meningococcal identified by qPCR. Results between specimen types were highly concordant for genogroups (Table 3).

**Table 3:** Prevalence of meningococcal MenACWY vaccine-type serogroups among OP and saliva samples collected from students (n = 299) and tested by qPCR.

Parameter	OP n (%) (*95% CI*)	Saliva n (%) (*95% CI*)	OP and saliva n (%) (*95% CI*)	Either OP or saliva n (%) (*95% CI*)	Concordance	*p* value*
menA	0	0	0	0	–	–
menB	25 (8.3) (*5.7–12.1*)	23 (7.7) (*5.2–11.3*)	22 (7.3) (*4.9–10.9*)	26 (8.7) (*6.0–12.4*)	98.7% (*96.6–99.5*)	0.6171
menC	2 (0.7) (*0.2–2.4*)	2 (0.7) (*0.2–2.4*)	2 (0.7) (*0.2–2.4*)	2 (0.7) (*0.2–2.4*)	100% (*98.7–100*)	–
menW	4 (1.3) (*0.5–3.4*)	3 (1.0) (*0.3–2.9*)	3 (1.0) (*0.3–2.9*)	4 (1.3) (*0.5–3.4*)	99.7% (*98.1–99.9*)	1.0000
menY	18 (6.0) (*3.8–9.3*)	14 (4.7) (*2.8–7.7*)	13 (4.3) (*2.6–7.3*)	19 (6.4) (*4.1–9.7*)	98.0% (*95.7–99.1*)	0.2207

*p values are calculated from McNemar tests comparing students positive for serogroup in oropharyngeal samples and saliva samples.

The percentage of concordance displays the proportion of samples (n = 63) with identical result in serogroup-specific qPCR assay for a particular serogroup.

## Discussion

In this cross-sectional study, we evaluated the application of saliva for meningococcal carriage detection using both culture and qPCR-based methods. Our goal was to optimize meningococcal detection for future carriage studies assessing the impact of the meningococcal vaccines on carriage. Although meningococcal detection with culture resulted in fewer meningococcal carriers identified in saliva when compared with oropharyngeal samples, qPCR detection of meningococcus did not result in significant differences between these two sample types.

Based on culture, we observed an overall carriage prevalence of 24.1%. The difference in positivity for meningococcus between oropharyngeal and saliva samples was likely caused by a greater difficulty to isolate meningococci from saliva cultures. In the saliva, a higher abundance of commensal species capable of growth on the culture media was observed and described first by Gordon in 1916^18^. Using qPCR detection we observed an overall carriage prevalence of 20.4%, and no significant differences were observed between oropharyngeal and saliva samples in positivity for meningococcus. Importantly, when detecting carriage of genogroupable meningococci, the method of testing culture-enriched saliva with a qPCR performed at least equally well compare with conventional diagnostic culture of oropharyngeal swab. Although numerous studies have implicated oral fluids in meningococcal transmission^8–16^, very few that tested saliva as specimen for assessing meningococcal carriage^18–20^ report on seemingly contradictory results. In this context, our findings are in line with a meningococcal carriage study conducted recently by Rodrigues et al.^20^ but in opposition to findings by Orr et al. describing a virtual absence of viable meningococci in saliva of *N. meningitidis* carriers^19^. However, with saliva collected by Orr et al. by swabbing gingiva, the volume of oral fluids cultured was likely to be lower than in our study, reducing sensitivity of carriage detection. Moreover, although we do not dispute the bactericidal properties of saliva, we believe that Gordon attributed the failure to culture *N. meningitidis* primarily to the highly polymicrobial nature of the sample rather than meningocidal properties of oral fluids per se^18^. This limitation can be addressed with the introduction of antibiotics as supplements in media selective for meningococci, and the application of qPCR detection methods^26^.

Among 299 students, we observed an overall meningococcal carriage rate of 24.7%, a prevalence that is in line to what has been reported previously with pharyngeal swabs for this age group^2,15^. The prevalence of meningococcal carriage among young adults is considered to be higher than other age groups due to increased social interactions which facilitate meningococcal transmission^6^. In addition, age-related alterations in the microbiota of the URT may prime individuals for meningococcal colonization^27^.

VT serogroups targeted in the MenACWY vaccine accounted for 41.0% of meningococci detected in carriage, corresponding to a prevalence of 8.4%. Of these VT genogroups, genogroup Y was most frequently detected. While an outbreak of serogroup W was ongoing in the Netherlands during the fall of 2018, the prevalence of genogroup W in carriage was low (1.3%). The prevalence of genogroup C was also low, possibly reflecting reduced circulation since implementation of menC vaccine in the Netherlands in 2002 that included catch-up for 1–18 year-olds^28^. Although we did not collect information on participants vaccination status, with an approximately 94% vaccination coverage in the teenagers in the Netherlands we assume the great majority of study participants had received the menC vaccine^28^. Serogroup A was not detected in our study, its circulation appears to be limited in the Netherlands^15,29^. The most prevalent genogroup among carriers was B. Genogroups B and Y have both been described to be most commonly detected genogroups among young adults^26^. The timing of our study coincided with start of a menACWY vaccine campaign among 14–18 years-olds^3^. However, at the time of our study individuals aged 16–18 years were not yet invited for the menACWY vaccine campaign, therefore we do not believe the menACWY vaccine had any substantial impact on the study findings.

Our study has a number of limitations. Firstly, MALDI-ToF may have identified more samples of students positive for meningococcus than qPCR detection with *metA* and *ctrA* carriage criterium as MALDI-ToF also takes non-genogroupable, unencapsulated meningococci into account, and is susceptible to misidentification^30^. To avoid misidentification by MALDI-ToF, we have only included bacterial strains for which identification displayed high confidence (≥ 2.0). Another limitation is false-positivity of qPCR tests. To minimize this issue, we have conducted ROC curve analysis and used the Youden index to determine a cut-off value for qPCR detection. Considering that the majority of qPCR positive samples facilitated successful recovery of viable meningococci, we conclude that false-positive results have had no significant impact on our conclusions.

One of the strengths of our study was the paired comparison of saliva and oropharyngeal samples in detection of meningococcal carriage. Furthermore, we have used selective media and inoculated plates immediately after samples collection. Fast processing of samples may be crucial for the sensitivity of meningococcal detection. Moreover, the combined use of two meningococcal qPCR targets for specific meningococcal detection in polymicrobial samples has allowed us to detect meningococcus with high specificity.

In conclusion, our findings show that the detected prevalence of meningococcal carriage between oropharyngeal and saliva samples was nondifferent with qPCR detection, the results for saliva were highly concordant with oropharyngeal swabs, and that the majority of samples positive with qPCR were shown to contain viable meningococci. Since the collection of saliva is easy, well tolerated and can be performed without professional assistance, we propose that saliva combined with qPCR-based surveillance can be considered for future meningococcal carriage studies.

## Supplementary Information

Supplementary Information.

## References

[1] Jafri RZ, Ali A, Messonnier NE (2013). Global epidemiology of invasive meningococcal disease. Popul. Health Metr..

[2] Christensen H, May M, Bowen L, Hickman M, Trotter CL (2010). Meningococcal carriage by age: A systematic review and meta-analysis. Lancet Infect. Dis..

[3] Knol MJ, Ruijs WL, Antonise-Kamp L, de Melker HE, van der Ende A (2018). Implementation of MenACWY vaccination because of ongoing increase in serogroup W invasive meningococcal disease, the Netherlands, 2018. Euro Surveill..

[4] Schurink-van 't Klooster, T. M. & de Melker, H. E. The National Immunisation Programme in the Netherlands: Surveillance and developments in 2018–2019 (Rijksinstituut voor Volksgezondheid en Milieu RIVM, 2019).

[5] Maiden MCJ, Ibarz-Pavón AB, Urwin R (2008). Impact of meningococcal serogroup C conjugate vaccines on carriage and herd immunity. J. Infect. Dis..

[6] Caugant DA, Maiden MC (2009). Meningococcal carriage and disease—Population biology and evolution. Vaccine.

[7] Roberts J, Greenwood B, Stuart J (2009). Sampling methods to detect carriage of *Neisseria**meningitidis*; literature review. J. Infect..

[8] Stanwell-Smith RE, Stuart JM, Hughes AO, Robinson P, Griffin MB, Cartwright K (1994). Smoking, the environment and meningococcal disease: A case control study. Epidemiol. Infect..

[9] Holdsworth G, Jackson H, Kaczmarski E (1996). Meningococcal infection from saliva. Lancet.

[10] Neal KR, Nguyen-Van-Tam JS, Jeffrey N (2000). Changing carriage rate of *Neisseria**meningitidis* among university students during the first week of term: Cross sectional study. BMJ.

[11] MacLennan J, Kafatos G, Neal K (2006). Social behavior and meningococcal carriage in British teenagers. Emerg. Infect. Dis..

[12] Tully J, Viner RM, Coen PG (2006). Risk and protective factors for meningococcal disease in adolescents: Matched cohort study. BMJ.

[13] Mandal S, Wu HM, MacNeil JR (2013). Prolonged university outbreak of meningococcal disease associated with a serogroup B strain rarely seen in the United States. Clin. Infect. Dis..

[14] Dryden AW, Rana M, Pandey P (2016). Primary meningococcal conjunctivitis: An unusual case of transmission by saliva. Digit. J. Ophthalmol..

[15] van Ravenhorst MB, Bijlsma MW, van Houten MA (2017). Meningococcal carriage in Dutch adolescents and young adults; A cross-sectional and longitudinal cohort study. Clin. Microbiol. Infect..

[16] McMillan M, Walters L, Mark T (2019). B Part of It study: A longitudinal study to assess carriage of *Neisseria**meningitidis* in first year university students in South Australia. Hum. Vaccin. Immunother..

[17] Nasidze I, Li J, Quinque D, Tang K, Stoneking M (2009). Global diversity in the human salivary microbiome. Genome Res..

[18] Gordon MH (1916). The inhibitory action of saliva on growth of the meningococcus. Br. Med. J..

[19] Orr HJ, Gray SJ, Macdonald M, Stuart JM (2003). Saliva and meningococcal transmission. Emerg. Infect. Dis..

[20] Rodrigues F, Christensen H, Morales-Aza B (2019). Viable *Neisseria**meningitidis* is commonly present in saliva in healthy young adults: Non-invasive sampling and enhanced sensitivity of detection in a follow-up carriage study in Portuguese students. PLoS One.

[21] Jordens JZ, Williams JN, Jones GR, Heckels JE (2002). Detection of meningococcal carriage by culture and PCR of throat swabs and mouth gargles. J. Clin. Microbiol..

[22] Miellet WR, van Veldhuizen J, Nicolaie MA (2021). Influenza-like illness exacerbates pneumococcal carriage in older adults. Clin. Infect. Dis..

[23] Diene SM, Bertelli C, Pillonel T (2016). Comparative genomics of *Neisseria meningitidis* strains: New targets for molecular diagnostics. Clin. Microbiol. Infect..

[24] Rojas E, Hoyos J, Oldfield NJ (2015). Optimization of molecular approaches to genogroup *Neisseria**meningitidis* carriage isolates and implications for monitoring the impact of new serogroup B vaccines. PLoS One.

[25] Nutz S, Doll K, Karlovsky P (2011). Determination of the LOQ in real-time PCR by receiver operating characteristic curve analysis: Application to qPCR assays for *Fusarium verticillioides* and *F. proliferatum*. Anal. Bioanal. Chem..

[26] Peterson ME, Li Y, Shanks H (2019). Serogroup-specific meningococcal carriage by age group: A systematic review and meta-analysis. BMJ Open.

[27] Moir JW (2015). Meningitis in adolescents: The role of commensal microbiota. Trends Microbiol..

[28] Kaaijk P, van der Ende A, Berbers G, van den Dobbelsteen GP, Rots NY (2012). Is a single dose of meningococcal serogroup C conjugate vaccine sufficient for protection? Experience from the Netherlands. BMC Infect. Dis..

[29] Bijlsma MW, Bekker V, Brouwer MC, Spanjaard L, van de Beek D, van der Ende A (2014). Epidemiology of invasive meningococcal disease in the Netherlands, 1960–2012: An analysis of national surveillance data. Lancet Infect. Dis..

[30] Cunningham SA, Mainella JM, Patel R (2014). Misidentification of *Neisseria**polysaccharea* as *Neisseria**meningitidis* with the use of matrix-assisted laser desorption ionization-time of flight mass spectrometry. J. Clin. Microbiol..

