# Supplementary material for: Detection of Neisseria meningitidis in saliva and oropharyngeal samples from college students
Source: Sci Rep. 2021 Nov 30;11:23138. doi: 10.1038/s41598-021-02555-x (PMC8632920; doi:10.1038/s41598-021-02555-x)
Supplement: Supplementary file 1 — Supplementary Information. [file 41598_2021_2555_MOESM1_ESM.pdf]

Supplementary Table S1 : Primer and probe concentrations used in the study.

Oligonucleotide	Sequence	Concentration (nM)	Reference
metA forward primer	5'-GCGAATTTGCTAATCCTATTTATGTGC-3'	750	Diene *et al* 2016
metA reverse primer	5'-AAATTTTGCGCCATTACAGGTG-3'	750	
metA probe	5'-6-FAM-AAATTTTGCGCCATTACAGGTG-3'-TAMRA	200	
ctrA forward primer	5'-TGGGCGGTTTGCAAGATC-3'	500	Rojas *et al* 2015
ctrA reverse primer	5'-TGACGTTCTGCCGGCAAT-3'	500	
ctrA probe	5'-6-FAM-CACACCACGCGCATCA -3'-TAMRA	200	
serogroup A (*csaB*) forward primer	5’-GCCACAAAGTGCCCTTCCT-3’	800	
serogroup A (*csaB*) forward primer	5’-TGGTATATGGTGCAAGCTGGTT-3’	800	
serogroup A (*csAB*) probe	5’-6-FAM-TTTAGCTCACATGCTATTG-3’-TAMRA	300	
serogroup B (*csB*) forward primer	5’-CCTCGGCTGGTAGTTATTAATGAAC-3’	300	
serogroup B (*csB*) reverse primer	5’-GCCAGGCCTATAATTCCTTTAGGA-3’	300	
serogroup B (*csB*) probe	5’-6-FAM-CCTTTTCTAATTGAGCCCCTAA-3’-TAMRA	100	
serogroup C (*csC*) forward primer	5’-GCACATTCAGGCGGGATTA-3’	200	
serogroup C (*csC*) reverse primer	5’-TTGAGATATGCGGTATTTGTCTTGA-3’	100	
serogroup C (*csC*) probe	5’-6-FAM-ACAAGCCAATCTATTGCT-3’-TAMRA	400	
serogroup W (*siaD*) forward primer	5’-CAGAAAGTGAGGGATTTCCATA-3’	200	
serogroup W (*siaD*) reverse primer	5’-CACAACCATTTTCATTATAGTTACTGT-3’	100	
serogroup W (*siaD*) probe	5’-6-FAM-TGGAAGGCATGGTGTATGATATTC-3’-TAMRA	100	
serogroup Y (*csy*) forward primer	5’-GTACGATATCCCTATCCTTGCCTATAA-3’	200	
serogroup Y (*csy*) reverse primer	5’-CCATTCCAGAAATATCACCAGTTTTA-3’	100	
serogroup Y (*csy*) probe	5’-6-FAM-TGGAGCGAATGATTTTAGCAA-3’-TAMRA	100	

Supplementary Table S2 : qPCR programmes used in this study.

qPCR assay	Step	Cycles	Temperature (°C)	Duration
metA and *ctrA* qPCR	Pre-incubation	1	95	10 min
	Denaturation	45	95	10 sec
	Annealing		60	45 sec
	Elongation		72	1 sec
serogroup-specific qPCR	Pre-incubation	1	95	5 min
	Denaturation	45	95	10 sec
	Annealing and elongation		60	50 sec

Supplementary Table S3 : *Neisseria meningitidis* strains used in this study.

Strain	Description	Source
serogroup A strain 3125	used to optimize the serogroup-specific qPCR assay	Meningococcal Reference Unit Manchester, UK
serogroup B strain BD00-00032	used to optimize the serogroup-specific qPCR assay	this study
serogroup C strain BD00-00268	used to optimize the serogroup-specific qPCR assay	this study
serogroup W strain BD98-00112	used to optimize the serogroup-specific qPCR assay, *ctrA* qPCR assay and *metA* qPCR assay.	this study
serogroup Y strain BD03-00373	used to optimize the serogroup-specific qPCR assay	this study

Supplementary Table S4 : Optimal qPCR C_T threshold and corresponding parameters for meningococcal carriage detection on samples stratified by positive or negative for culture detection.

Parameter	Optimal threshold *(95% CI)*	Youden index (J)	Sensitivity	Specificity
**OP *metA***	24.89 *(22.86 – 31.27)*	0.92	0.96	0.96
**OP *ctrA***	35.75 *(26.13 – 38.06)*	0.82	0.89	0.93
**Saliva *metA***	24.91 *(22.86 – 31.27)*	0.92	0.96	0.96
**Saliva *ctrA***	35.10 *(26.30 – 38.00)*	0.82	0.89	0.93

CI: confidence interval.

For qPCR detection of *Neisseria meningitidis,* we regarded a culture-enriched sample as positive by qPCR when detection of both the *metA* and *ctrA* genes was observed. For both types of samples and in both qPCRs we observed a bimodal distribution of C_Ts, with the highest C_T of any culture-positive sample separated by at least 10 C_Ts from the lowest in a cluster of all culture-negative samples. Based on this distribution, we performed ROC curve analysis to calculate the maximal Youden indices. For *metA*, the difference between thresholds for positivity calculated for oropharyngeal and saliva samples was within 0.1 C_T, for *ctrA* the difference was within one C_T. The difference between thresholds for *metA* and *ctrA* was over 10 C_Ts due to presence of non-genogroupable strains cultured that were likely to be *metA* positive yet *ctrA* negative. A criterium based on both *ctrA* and *metA* was expected to impact negatively the sensitivity of meningococcal carriage detection by qPCR when compared with culture due to presence of non-genogroupable meningococci that were likely to be *ctrA*-negative, therefore we applied tresholds (<25 C_T) calculated for *metA* also to *ctrA*.

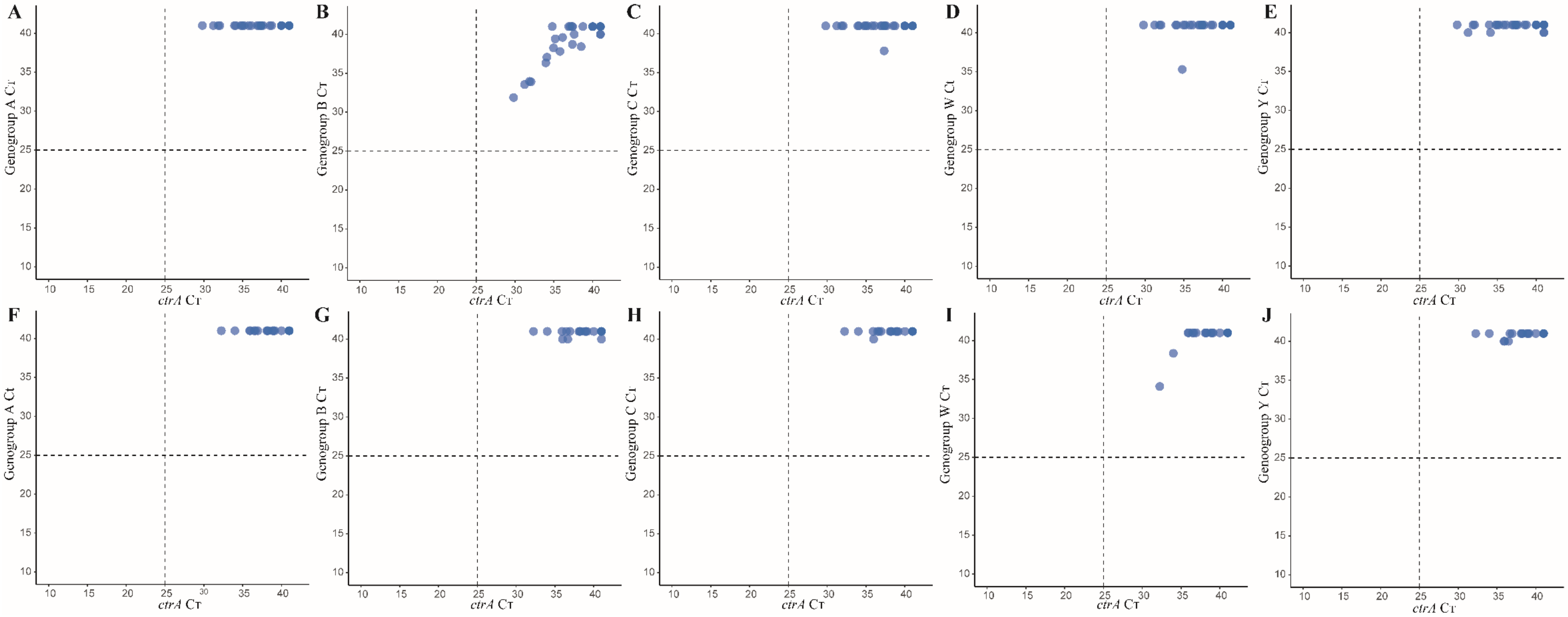

Figure S1. Scatterplots of genogroup-specific qPCR assays for CE samples negative for meningococcus by qPCR. Scatterplots displays genogroups-specific qPCR results for culture-enriched oropharyngeal (**A** – **E**) and saliva (**F** – **J**) samples negative for meningococcal carriage by qPCR and culture (n=42 for each). None of the tested samples generated a signal (C_T) below 25 C_T for any of the tested genogroups, namely serogroup A, B, C, W and Y. Dashed lines depict the C_T criterium for meningococcal carriage.
